# The generalized viscosity explicit rules for a family of strictly pseudo-contractive mappings in a *q*-uniformly smooth Banach space

**DOI:** 10.1186/s13660-018-1760-5

**Published:** 2018-07-11

**Authors:** Wongvisarut Khuangsatung, Pongsakorn Sunthrayuth

**Affiliations:** Department of Mathematics and Computer Science, Faculty of Science and Technology, Rajamangala University of Technology Thanyaburi (RMUTT), Pathumthani, Thailand

**Keywords:** Strict pseudo-contractions, Banach space, Strong convergence, Fixed point problem, Iterative method

## Abstract

In this paper, we construct an iterative method by a generalized viscosity explicit rule for a countable family of strictly pseudo-contractive mappings in a *q*-uniformly smooth Banach space. We prove strong convergence theorems of proposed algorithm under some mild assumption on control conditions. We apply our results to the common fixed point problem of convex combination of family of mappings and zeros of accretive operator in Banach spaces. Furthermore, we also give some numerical examples to support our main results.

## Introduction

In this paper, we assume that *E* is a real Banach space with dual space $E^{*}$ and *C* is a nonempty subset of *E*. Let $q > 1$ be a real number. The *generalized duality mapping* $J_{q} : E\to 2^{E^{*}}$ is defined by

$$ J_{q}(x)=\bigl\{ \bar{x}\in E^{*}:\langle x,\bar{x}\rangle= \Vert x \Vert ^{q}, \Vert \bar{x} \Vert = \Vert x \Vert ^{q-1} \bigr\} , $$

where $\langle\cdot,\cdot\rangle$ denotes the generalized duality pairing between elements of *E* and $E^{*}$. In particular, $J_{q}=J_{2}$ is called the *normalized duality mapping*. If *E* is smooth, then $J_{q}$ is single-valued and denoted by $j_{q}$ (see [1]). If $E:=H$ is a real Hilbert space, then $J=I$, where *I* is the identity mapping. Further, we have the following properties of the generalized duality mapping $J_{q}$:

- $J_{q}(x)=\|x\|^{q-2}J_{2}(x)$ for all $x\in E$ with $x\neq 0$.
- $J(tx)=t^{q-1}J_{q}(x)$ for all $x\in E$ and $t\geq 0$.
- $J_{q}(-x)=-J_{q}(x)$ for all $x\in E$.

Let *T* be a self-mapping of *C*. We denote the fixed point set of the mapping *T* by $F(T)=\{x\in C:x=Tx\}$. A mapping $f : C \to C$ is said to be a *contraction* if there exists a constant $\rho\in(0,1)$ satisfying

$$\begin{aligned} \bigl\Vert f(x)-f(y) \bigr\Vert \leq\rho \Vert x-y \Vert ,\quad \forall x,y\in C. \end{aligned}$$

We use $\Pi_{C}$ to denote the collection of all contractions from *C* into itself. Recall that a mapping $T:C\to C$ is said to be *nonexpansive* if

$$\begin{aligned} \Vert Tx-Ty \Vert \leq \Vert x-y \Vert ,\quad \forall x,y\in C. \end{aligned}$$

A mapping $T:C\to C$ is said to be *λ-strict pseudo-contraction* if for all $x,y\in C$, there exist $\lambda>0$ and $j_{q}(x-y)\in J_{q}(x-y)$ such that

1 $$\begin{aligned} \bigl\langle Tx-Ty,j_{q}(x-y)\bigr\rangle \leq \Vert x-y \Vert ^{q}-\lambda \bigl\Vert (I-T)x-(I-T)y \bigr\Vert ^{q},\quad \forall x,y\in C. \end{aligned}$$

It is not hard to show that (1) equivalent to the following inequality:

2 $$\begin{aligned} \bigl\langle (I-T)x-(I-T)y,j_{q}(x-y)\bigr\rangle \geq \lambda \bigl\Vert (I-T)x-(I-T)y \bigr\Vert ^{q},\quad \forall x,y \in C. \end{aligned}$$

If $E:=H$ is a Hilbert space, then (1) (and so (2)) is equivalent to the following inequality:

3 $$\begin{aligned} \Vert Tx-Ty \Vert ^{2}\leq \Vert x-y \Vert ^{2}+k \bigl\Vert (I-T)x-(I-T)y \bigr\Vert ^{2},\quad \forall x,y\in C, \end{aligned}$$

where $k=1-2\lambda<1$. We assume that $k\geq0$, so that $k\in[0,1)$. Note that the class of strictly pseudo-contractive mappings include the class of nonexpansive mappings as a particular case in Hilbert spaces. Clearly, *T* is nonexpansive if and only if *T* is a 0-strict pseudo-contraction. Strict pseudo-contractions were first introduced by Browder and Petryshyn [2] in 1967. They have more powerful applications than nonexpansive mappings do in solving inverse problems (see, e.g., [3]). Therefore it is more interesting to study the theory of iterative methods for strictly pseudo-contractive mappings. Several researchers studied the class of strictly pseudo-contractive mappings in Hilbert and Banach spaces (see, e.g., [4–9] and the references therein).

Now, we give some examples of *λ*-strictly pseudo-contractive mappings.

### Example 1.1

([8])

Let $E=\mathbb{R}$ with the usual norm, and let $C=(0,\infty)$. Let $T:C\to C$ be defined by

$$Tx=\frac{x^{2}}{1+x},\quad x\in C. $$

Then, *T* is a 1-strict pseudo-contraction.

### Example 1.2

([8])

Let $E=\mathbb{R}$ with the usual norm, and let $C=[-1,1]$. Let $T:C\to C$ be defined by

$$ Tx=\textstyle\begin{cases} x,&x\in[-1,0],\\ x-x^{2},&x\in[0,1]. \end{cases} $$

Then, *T* is a *λ*-strict pseudo-contraction with constant $\lambda>0$.

Over the last several years, the implicit midpoint rule (IMR) has become a powerful numerical method for numerically solving time-dependent differential equations (in particular, stiff equations) (see [10–15]) and differential algebraic equations (see [16]). Consider the following initial value problem:

4 $$\begin{aligned} x'(t)=f \bigl(x(t) \bigr),\quad x(t_{0})=x_{0}, \end{aligned}$$

where $f:\mathbb{R}^{M}\to\mathbb{R}^{M}$ is a continuous function. The IMR is an implicit method given by the following finite difference scheme [17]:

5 $$ \textstyle\begin{cases} y_{0}=x_{0},\\ y_{n+1}=y_{n}+hf (\frac{y_{n}+y_{n+1}}{2} ),\quad n\geq0, \end{cases} $$

where $h>0$ is a time step. It is known that if $f:\mathbb{R}^{M}\to\mathbb{R}^{M}$ is Lipschitz continuous and sufficiently smooth, then the sequence $\{y_{n}\}$ converges to the exact solution of (4) as $h\to0$ uniformly over $t\in[t_{0},t^{*}]$ for any fixed $t^{*}>0$. If the function *f* is written as $f(x) = x - g(x)$, then (5) becomes

6 $$ \textstyle\begin{cases} y_{0}=x_{0},\\ y_{n+1}=y_{n}+h [\frac{y_{n}+y_{n+1}}{2}-g (\frac{y_{n}+y_{n+1}}{2} ) ],\quad n\geq0, \end{cases} $$

and the critical points of (4) are the fixed points of the problem $x = g(x)$.

Based on IMR (5), Alghamdi et al. [18] introduced the following two algorithms for the solution of the fixed point problem $x=Tx$, where *T* is a nonexpansive mapping in a Hilbert space *H*:

7 $$\begin{aligned} &x_{n+1}=x_{n}-t_{n} \biggl[\frac{x_{n}+x_{n+1}}{2}-T \biggl(\frac{x_{n}+x_{n+1}}{2} \biggr) \biggr],\quad n\geq0, \end{aligned}$$

8 $$\begin{aligned} &x_{n+1}=(1-t_{n})x_{n}+t_{n}T \biggl( \frac{x_{n}+x_{n+1}}{2} \biggr),\quad n\geq0, \end{aligned}$$

for $x_{0}\in H$, with $\{t_{n}\}_{n=1}^{\infty}\subset(0,1)$. They proved that these two schemes converge weakly to a point in $F(T)$.

To obtain strong convergence, Xu et al. [19] applied the viscosity approximation method introduced by Moudafi [20] to the IMR for a nonexpansive mapping *T* and proposed the following *viscosity implicit midpoint rule* in Hilbert spaces *H* as follows:

9 $$ x_{n+1}=\alpha_{n}f(x_{n})+(1- \alpha_{n})T \biggl(\frac{x_{n}+x_{n+1}}{2} \biggr),\quad n\geq1, $$

where $\{\alpha_{n}\}$ is a real control condition in $(0,1)$. They also proved that the sequence $\{x_{n}\}$ generated by (9) converges strongly to a point $x^{*}\in F(T)$, which solves the variational inequality

10 $$ \bigl\langle (f-I)x^{*},z-x^{*}\bigr\rangle \leq0,\quad z\in F(T). $$

Later, Ke and Ma [21] improved the viscosity implicit midpoint rule by replacing the midpoint by any point of the interval $[x_{n},x_{n+1}]$. They introduced the so-called *generalized viscosity implicit rules* to approximating the fixed point of a nonexpansive mapping *T* in Hilbert spaces *H* as follows:

11 $$ x_{n+1}=\alpha_{n}f(x_{n})+(1- \alpha_{n})T\bigl(s_{n}x_{n}+(1-s_{n})x_{n+1} \bigr),\quad n\geq1. $$

They also proved that the sequence $\{x_{n}\}$ generated by (11) converges strongly to a point $x^{*}\in F(T)$ that solves the variational inequality (10).

In numerical analysis, it is clear that the computation by the IMR is not an easy work in practice. Because the IMR need to compute at every time steps, it can be much harder to implement. To overcome this difficulty, for solving (4), we consider the helpful method, the so-called *explicit midpoint method* (EMR), given by the following finite difference scheme [22, 23]:

12 $$ \textstyle\begin{cases} y_{0}=x_{0},\\ \bar{y}_{n+1}=y_{n}+hf(y_{n}),\\ y_{n+1}=y_{n}+hf (\frac{y_{n}+\bar{y}_{n+1}}{2} ),\quad n\geq0. \end{cases} $$

Note that the EMR (12) calculates the system status at a future time from the currently known system status, whereas IMR (5) calculates the system status involving both the current state of the system and the later one (see [23, 24]).

In 2017, Marino et al. [25] combined the generalized viscosity implicit midpoint rules (11) with the EMR (12) for a quasi-nonexpansive mapping *T* and introduced the following so-called *generalized viscosity explicit midpoint rule* in Hilbert spaces *H* as follows:

13 $$ \textstyle\begin{cases} \bar{x}_{n+1}=\beta_{n}x_{n}+(1-\beta_{n})Tx_{n},\\ x_{n+1}=\alpha_{n} f(x_{n})+(1-\alpha_{n})T(s_{n}x_{n}+(1-s_{n})\bar{x}_{n+1}),\quad n\geq1. \end{cases} $$

They also showed that, under certain assumptions imposed on the parameters, the sequence $\{x_{n}\}$ generated by (13) converges strongly to a point $x^{*}\in F(T)$, which solves the variational inequality (10).

The above results naturally bring us to the following questions.

### Question 1

Can we extend the generalized viscosity explicit midpoint rule (13) to higher spaces other than Hilbert spaces? Such as a 2-uniformly smooth Banach space or, more generally, in a *q*-uniformly smooth Banach space.

### Question 2

Can we obtain a strong convergence result of generalized viscosity explicit midpoint rule (13) for finding the set of common fixed points of a family of mappings? Such as a countable family of strict pseudo-contractions.

The purpose of this paper is to give some affirmative answers to the questions raised. We introduce an iterative algorithm for finding the set of common fixed points of a countable family of strict pseudo-contractions by a generalized viscosity explicit rule in a *q*-uniformly smooth Banach space. We prove the strong convergence of the proposed algorithm under some mild assumption on control conditions. We apply our results to the common fixed point problem of a convex combination of a family of mappings and zeros of an accretive operator in Banach spaces. Furthermore, we also give some numerical examples to support our main results.

## Preliminaries

Let *E* be a real Banach space with norm $\|\cdot\|$ and dual space $E^{*}$ of *E*. The symbol $\langle x,x^{*}\rangle$ denotes the pairing between *E* and $E^{*}$, that is, $\langle x,x^{*}\rangle=x^{*}(x)$, the value of $x^{*}$ at *x*. The *modulus of convexity* of *E* is the function $\delta:(0,2]\to[0,1]$ defined by

$$ \delta(\epsilon)=\inf \biggl\{ 1-\frac{ \Vert x+y \Vert }{2}:x,y\in E, \Vert x \Vert = \Vert y \Vert =1, \Vert x-y \Vert \geq\epsilon \biggr\} . $$

A Banach space *E* is said to be *uniformly convex* if $\delta_{E}(\epsilon)>0$ for all $\epsilon\in (0,2]$. For $p > 1$, we say that *E* is said to be *p-uniformly convex* if there is $c_{p} > 0$ such that $\delta_{E}(\epsilon)\geq c_{p}\epsilon^{p}$ for all $\epsilon\in (0,2]$.

The *modulus of smoothness* of *E* is the function $\rho_{E}:\mathbb{R}^{+}:=[0,\infty)\to\mathbb{R}^{+}$ defined by

$$ \rho_{E}(\tau)=\sup \biggl\{ \frac{ \Vert x+\tau y \Vert + \Vert x- \tau y \Vert }{2}-1: \Vert x \Vert , \Vert y \Vert \leq1 \biggr\} . $$

A Banach space *E* is said to be *uniformly smooth* if $\frac{\rho_{E}(\tau)}{\tau}\to 0$ as $\tau\to 0$. For $q>1$, a Banach space *E* is said to be *q-uniformly smooth* if there exists $c_{q}> 0$ such that $\rho_{E}(\tau)\leq c_{q}\tau^{q}$ for all $\tau>0$. If *E* is *q*-uniformly smooth, then $q\leq2$, and *E* is also uniformly smooth. Further, *E* is *p*-uniformly convex (*q*-uniformly smooth) if and only if $E^{*}$ is *q*-uniformly smooth (*p*-uniformly convex), where $p\geq2$ and $1 < q\leq 2$ satisfy $\frac{1}{p}+\frac{1}{q}=1$. It is well known that Hilbert spaces $L_{p}$ and $l_{p}$ $(p > 1)$ are uniformly smooth (see [26]). More precisely, the spaces $L_{p}$ and $l_{p}$ are $\min\{p,2\}$-uniformly smooth for every $p > 1$.

### Definition 2.1

Let *C* a be nonempty closed convex subsets of *E*, and let *Q* be a mapping of *E* onto *C*. Then *Q* is said to be:

- *sunny* if $Q(Qx+t(x-Qx))=Qx$ for all $x\in C$ and $t\geq 0$.
- *retraction* if $Qx=x$ for all $x\in C$.
- a sunny nonexpansive retraction if *Q* is sunny, nonexpansive, and a retraction from *E* onto *C*.

It is known that if $E:=H$ is a real Hilbert space, then a sunny nonexpansive retraction *Q* coincides with the metric projection from *E* onto *C*. Moreover, if *E* is uniformly smooth and *T* is a nonexpansive mapping of *C* into itself with $F(T)\neq\emptyset$, then $F(T)$ is a sunny nonexpansive retraction from *E* onto *C* (see [27]). We know that in a uniformly smooth Banach space, a retraction $Q : C \to E$ is sunny and nonexpansive if and only if $\langle x-Qx,j_{q}(y-Qx)\rangle\leq0$ for all $x\in E$ and $y\in C$ (see [28]).

### Lemma 2.2

([29])

*Let* *C* *be a nonempty closed convex subset of a uniformly smooth Banach space* *E*. *Let* $S:C\to C$ *be a nonexpansive self*-*mapping such that* $F(S)\neq\emptyset$ *and* $f\in \Pi_{C}$. *Let* $\{z_{t}\}$ *be the net sequence defined by*

$$\begin{aligned} z_{t}=tf(z_{t})+(1-t)Sz_{t},\quad t\in(0,1). \end{aligned}$$

*Then*:

(i) $\{x_{t}\}$ *converges strongly as* $t\to0$ *to a point* $Q(f)\in F(S)$, *which solves the variational inequality*
  $$\begin{aligned} \bigl\langle (I-f)Q(f),j_{q}\bigl(Q(f)-z\bigr)\bigr\rangle \leq0,\quad z\in F(S). \end{aligned}$$
(ii) *Suppose that* $\{x_{n}\}$ *is a bounded sequence such that* $\lim_{n\to\infty}\|x_{n}-Sx_{n}\|=0$. *If* $Q(f):=\lim_{t\to 0}x_{t}$ *exists*, *then*
  $$\begin{aligned} \limsup_{n\to\infty}\bigl\langle (f-I)Q(f),j_{q} \bigl(x_{n}-Q(f)\bigr)\bigr\rangle \leq0. \end{aligned}$$

### Lemma 2.3

([30])

*Let* *C* *be a nonempty closed convex subset of a real* *q*-*uniformly smooth Banach space* *E*. *Let* $T : C \to C$ *be a* *λ*-*strict pseudo*-*contraction*. *For all* $x\in C$, *we define* $T_{\theta}x:=(1-\theta)x+\theta Tx$. *Then*, *as* $\theta\in (0,\delta]$, $\delta=\min \{1, (\frac{q\lambda}{\kappa_{q}} )^{\frac{1}{q-1}} \}$, *where* $\kappa_{q}$ *is the* *q*-*uniform smoothness constant*, *and* $T_{\theta}:C\to C$ *is nonexpansive such that* $F(T_{\theta}) = F(T)$.

Using the concept of subdifferentials, we have the following inequality.

### Lemma 2.4

([31])

*Let* $q > 1$, *and let* *E* *be a real normed space with the generalized duality mapping* $J_{q}$. *Then*, *for any* $x, y \in E$, *we have*

14 $$\begin{aligned} \Vert x+y \Vert ^{q}\leq \Vert x \Vert ^{q}+q\bigl\langle y,j_{q}(x+y)\bigr\rangle , \end{aligned}$$

*where* $j_{q}(x+y)\in J_{q}(x+y)$.

### Lemma 2.5

([32])

*Let* $p > 1$ *and* $r > 0$ *be two fixed real numbers*, *and let* *E* *be a uniformly convex Banach space*. *Then*, *for all* $x,y\in B_{r}$ *and* $t\in[0,1]$,

$$\begin{aligned} \bigl\Vert tx+(1-t)y \bigr\Vert ^{p}\leq t \Vert x \Vert ^{p}+(1-t) \Vert y \Vert ^{p}-t(1-t)c \Vert x-y \Vert ^{p}, \end{aligned}$$

*where* $c>0$.

### Lemma 2.6

([33])

*Suppose that* $q > 1$. *Then*

$$ ab\leq\frac{1}{q}a^{q}+ \biggl(\frac{q-1}{q} \biggr)b^{\frac{q}{q-1}} $$

*for positive real numbers* $a, b$.

### Lemma 2.7

([34])

*Let* $\{a_{n}\}$ *be a sequence of nonnegative real numbers*, $\{\gamma_{n}\}$ *be a sequence of* $(0,1)$ *with* $\sum_{n=1}^{\infty}\gamma_{n}=\infty$, $\{c_{n}\}$ *be a sequence of nonnegative real number with* $\sum_{n=1}^{\infty}c_{n}<\infty$, *and let* $\{b_{n}\}$ *be a sequence of real numbers with* $\limsup_{n\to\infty}b_{n}\leq0$. *Suppose that*

$$\begin{aligned} a_{n+1}=(1-\gamma_{n})a_{n}+\gamma_{n} b_{n} +c_{n} \end{aligned}$$

*for all* $n\in\mathbb{N}$. *Then*, $\lim_{n\to\infty}a_{n}=0$.

### Lemma 2.8

([35])

*Let* $\{s_{n}\}$ *be sequences of real numbers such that there exists a subsequence* $\{n_{i}\}$ *of* $\{n\}$ *such that* $s_{n_{i}}< s_{{n_{i}}+1}$ *for all* $i\in\mathbb{N}$. *Then there exists an increasing sequence* $\{m_{k}\}\subset\mathbb{N}$ *such that* $\lim_{k\to\infty}m_{k}=\infty$ *and the following properties are satisfied by all sufficiently large numbers* $k\in\mathbb{N}$:

$$\begin{aligned} s_{m_{k}}\leq s_{{m_{k}}+1}\quad \textit{and}\quad s_{k}\leq s_{{m_{k}}+1}. \end{aligned}$$

*In fact*, $m_{k}:=\max\{j\leq k:s_{j}\leq s_{j+1}\}$.

### Definition 2.9

([34])

Let *C* be a nonempty closed convex subset of a real Banach space *E*. Let $\{T_{n}\}_{n=1}^{\infty}$ be a family of mappings of *C* into itself. We say that $\{T_{n}\}_{n=1}^{\infty}$ satisfies the $AKTT$-condition if

15 $$ \sum_{n=1}^{\infty}\sup _{w\in C} \Vert T_{n+1}w-T_{n}w \Vert < \infty. $$

### Lemma 2.10

([34])

*Let* *C* *be a nonempty closed convex subset of a real Banach space* *E*. *Suppose that* $\{T_{n}\}_{n=1}^{\infty}$ *satisfies the AKTT*-*condition*. *Then*, *for each* $x\in C$, $\{T_{n}x\}$ *converges strongly to some point of* *C*. *Moreover*, *let* *T* *be the mapping of* *C* *into itself defined by* $Tx=\lim_{n\to\infty}T_{n}x$ *for all* $x\in C$. *Then*, $\lim_{n\to\infty}\sup_{w\in C}\|Tw-T_{n}w\|=0$.

In the following, we will write that $(\{T_{n}\}, T )$ satisfies the *AKTT*-condition if $\{T_{n}\}$ satisfies the *AKTT*-condition and *T* is defined by Lemma 2.10 with $F(T)=\bigcap_{n=1}^{\infty}F(T_{n})$.

## Main results

### Theorem 3.1

*Let* *C* *be a nonempty closed convex subset of a real uniformly convex and* *q*-*uniformly smooth Banach space* *E*. *Let* $f\in\Pi_{C}$ *with coefficient* $\rho\in(0,1)$, *and let* $\{T_{n}\}_{n=1}^{\infty}:C\to C$ *be a family of* *λ*-*strict pseudo*-*contractions such that* $\Omega:=\bigcap_{n=1}^{\infty}F(T_{n})\neq\emptyset$. *For all* $x\in C$, *define the mapping* $S_{n}x=(1-\theta_{n})x+\theta_{n} T_{n}x$, *where* $0<\theta_{n}\leq\delta$, $\delta=\min \{1, (\frac{q\lambda}{\kappa_{q}} )^{\frac{1}{q-1}} \}$, *and* $\sum_{n=1}^{\infty}|\theta_{n+1}-\theta_{n}|<\infty$. *For given* $x_{1}\in C$, *let* $\{x_{n}\}$ *be a sequence generated by*

16 $$ \textstyle\begin{cases} \bar{x}_{n+1}=\beta_{n}x_{n}+(1-\beta_{n})S_{n}x_{n},\\ x_{n+1}=\alpha_{n} f(x_{n})+(1-\alpha_{n})S_{n}(t_{n}x_{n}+(1-t_{n})\bar{x}_{n+1}),\quad n\geq1, \end{cases} $$

*where* $\{\alpha_{n}\}$, $\{\beta_{n}\}$, *and* $\{t_{n}\}$ *are sequences in* $(0,1)$ *satisfying the following conditions*:

1. $\lim_{n\to\infty}\alpha_{n}=0$, $\sum_{n=1 }^{\infty}\alpha_{n}=\infty$;
2. $\liminf_{n\to\infty}\beta_{n}(1-\beta_{n})(1-t_{n})>0$.

*Suppose in addition that* $(\{T_{n}\}_{n=1}^{\infty},T)$ *satisfies the* $AKTT$-*condition*. *Then*, $\{x_{n}\}$ *defined by* (16) *converges strongly to* $x^{*}=Q(f)\in\Omega$, *which solves the variational inequality*

17 $$\begin{aligned} \bigl\langle (I-f)Q(f),j_{q}\bigl(Q(f)-z\bigr)\bigr\rangle \leq0,\quad z\in \Omega, \end{aligned}$$

*where* *Q* *is a sunny nonexpansive retraction of* *C* *onto* Ω.

### Proof

First, we show that $\{x_{n}\}$ is bounded. From Lemma 2.3 we have that $S_{n}$ is nonexpansive such that $F(S_{n})=F(T_{n})$ for all $n\geq1$. Put $z_{n}:=t_{n}x_{n}+(1-t_{n})\bar{x}_{n+1}$. For each $z\in\Omega:=\bigcap_{n=1}^{\infty}F(T_{n})$, we have

18 $$\begin{aligned} \Vert z_{n}-z \Vert =& \bigl\Vert t_{n}(x_{n}-z)+(1-t_{n}) (\bar{x}_{n+1}-z) \bigr\Vert \\ \leq&t_{n} \Vert x_{n}-z \Vert +(1-t_{n}) \Vert \bar{x}_{n+1}-z \Vert \\ \leq&t_{n} \Vert x_{n}-z \Vert +(1-t_{n}) \bigl(\beta_{n} \Vert x_{n}-z \Vert +(1- \beta_{n}) \Vert S_{n}x_{n}-z \Vert \bigr) \\ \leq&t_{n} \Vert x_{n}-z \Vert +(1-t_{n}) \beta_{n} \Vert x_{n}-z \Vert +(1-t_{n}) (1- \beta_{n}) \Vert x_{n}-z \Vert \\ =& \Vert x_{n}-z \Vert . \end{aligned}$$

It follows that

$$\begin{aligned} \Vert x_{n+1}-z \Vert =& \bigl\Vert \alpha_{n}f(x_{n})+(1- \alpha_{n})S_{n}z_{n}-z \bigr\Vert \\ =& \bigl\Vert \alpha_{n}\bigl(f(x_{n})-f(z)\bigr)+ \alpha_{n}\bigl(f(z)-z\bigr)+(1-\alpha_{n}) (S_{n}z_{n}-z) \bigr\Vert \\ \leq&\alpha_{n} \bigl\Vert f(x_{n})-f(z) \bigr\Vert + \alpha_{n} \bigl\Vert f(z)-z \bigr\Vert +(1-\alpha_{n}) \Vert S_{n}z_{n}-z \Vert \\ \leq&\bigl(1-(1-\rho)\alpha_{n}\bigr) \Vert x_{n}-z \Vert +(1-\rho)\alpha_{n}\frac{ \Vert f(z)-z \Vert }{1-\rho} \\ \leq&\max \biggl\{ \Vert x_{n}-z \Vert ,\frac{ \Vert f(z)-z \Vert }{1-\rho} \biggr\} . \end{aligned}$$

By induction we have

$$\begin{aligned} \Vert x_{n}-z \Vert \leq&\max \biggl\{ \Vert x_{1}-z \Vert ,\frac{ \Vert f(z)-z \Vert }{1-\rho} \biggr\} ,\quad n\geq1. \end{aligned}$$

Hence $\{x_{n}\}$ is bounded. Consequently, we deduce immediately that $\{f(x_{n})\}$ and $\{S_{n}(t_{n}x_{n}+(1-t_{n})\bar{x}_{n+1})\}$ are bonded. Let $x^{*}=Q(f)$. By the convexity of $\|\cdot\|^{q}$ and Lemma 2.5 we have

19 $$\begin{aligned} \bigl\Vert S_{n}z_{n}-x^{*} \bigr\Vert ^{q} \leq& \bigl\Vert z_{n}-x^{*} \bigr\Vert ^{q} \\ =& \bigl\Vert t_{n}\bigl(x_{n}-x^{*}\bigr)+(1-t_{n}) \bigl(\bar{x}_{n+1}-x^{*}\bigr) \bigr\Vert ^{q} \\ \leq&t_{n} \bigl\Vert x_{n}-x^{*} \bigr\Vert ^{q}+(1-t_{n}) \bigl\Vert \bar{x}_{n+1}-x^{*} \bigr\Vert ^{q} \\ =&t_{n} \bigl\Vert x_{n}-x^{*} \bigr\Vert ^{q}+(1-t_{n}) \bigl\Vert \beta_{n} \bigl(x_{n}-x^{*}\bigr)+(1-\beta_{n}) \bigl(S_{n}x_{n}-x^{*} \bigr) \bigr\Vert ^{q} \\ \leq&t_{n} \bigl\Vert x_{n}-x^{*} \bigr\Vert ^{q}+(1-t_{n}) \bigl[\beta_{n} \bigl\Vert x_{n}-x^{*} \bigr\Vert ^{q}+(1-\beta_{n}) \bigl\Vert S_{n}x_{n}-x^{*} \bigr\Vert ^{q} \\ &{}- \beta_{n}(1-\beta_{n})c \Vert x_{n}-S_{n}x_{n} \Vert ^{q} \bigr] \\ \leq& \bigl\Vert x_{n}-x^{*} \bigr\Vert ^{q}- \beta_{n}(1-\beta_{n}) (1-t_{n})c \Vert x_{n}-S_{n}x_{n} \Vert ^{q}. \end{aligned}$$

It follows from Lemma 2.4 and (19) that

20 $$\begin{aligned} & \bigl\Vert x_{n+1}-x^{*} \bigr\Vert ^{q} \\ &\quad = \bigl\Vert \alpha_{n}\bigl(f(x_{n})-x^{*}\bigr)+(1- \alpha_{n}) \bigl(S_{n}z_{n}-x^{*}\bigr) \bigr\Vert ^{q} \\ &\quad = \bigl\Vert \alpha_{n}\bigl(f(x_{n})-f\bigl(x^{*}\bigr) \bigr)+\alpha_{n}\bigl(f\bigl(x^{*}\bigr)-x^{*}\bigr)+(1- \alpha_{n}) \bigl(S_{n}z_{n}-x^{*}\bigr) \bigr\Vert ^{q} \\ &\quad \leq \bigl\Vert \alpha_{n}\bigl(f(x_{n})-f\bigl(x^{*} \bigr)\bigr)+(1-\alpha_{n}) \bigl(S_{n}z_{n}-x^{*} \bigr) \bigr\Vert ^{q}+q\alpha_{n}\bigl\langle f\bigl(x^{*} \bigr)-x^{*},j_{q}\bigl(x_{n+1}-x^{*}\bigr)\bigr\rangle \\ &\quad \leq\alpha_{n} \bigl\Vert f(x_{n})-f\bigl(x^{*}\bigr) \bigr\Vert ^{q}+(1-\alpha_{n}) \bigl\Vert S_{n}z_{n}-x^{*} \bigr\Vert ^{q}+q \alpha_{n}\bigl\langle f\bigl(x^{*}\bigr)-x^{*},j_{q} \bigl(x_{n+1}-x^{*}\bigr)\bigr\rangle \\ &\quad \leq \alpha_{n} \bigl\Vert f(x_{n})-f\bigl(x^{*}\bigr) \bigr\Vert ^{q}+(1-\alpha_{n}) \bigl[ \bigl\Vert x_{n}-x^{*} \bigr\Vert ^{q}-\beta_{n}(1- \beta_{n}) (1-t_{n})c \Vert x_{n}-S_{n}x_{n} \Vert ^{q} \bigr] \\ &\qquad {}+q\alpha_{n}\bigl\langle f\bigl(x^{*}\bigr)-x^{*},j_{q} \bigl(x_{n+1}-x^{*}\bigr)\bigr\rangle \\ &\quad \leq \bigl(1-(1-\rho)\alpha_{n}\bigr) \bigl\Vert x_{n}-x^{*} \bigr\Vert ^{q}-(1-\alpha_{n}) \beta_{n}(1-\beta_{n}) (1-t_{n})c \Vert x_{n}-S_{n}x_{n} \Vert ^{q} \\ &\qquad {}+q\alpha_{n}\bigl\langle f\bigl(x^{*}\bigr)-x^{*},j_{q} \bigl(x_{n+1}-x^{*}\bigr)\bigr\rangle . \end{aligned}$$

The rest of the proof will be divided into two cases:

*Case 1*. Suppose that there exists $n_{0}\in\mathbb{N}$ such that $\{\|x_{n}-x^{*}\|\}_{n=n_{0}}^{\infty}$ is nonincreasing. This implies that $\{\|x_{n}-x^{*}\|\}_{n=1}^{\infty}$ is convergent. From (20) we see that

$$\begin{aligned} (1-\alpha_{n})\beta_{n}(1-\beta_{n}) (1-s_{n})c \Vert x_{n}-S_{n}x_{n} \Vert ^{q}\leq \bigl\Vert x_{n}-x^{*} \bigr\Vert ^{q}- \bigl\Vert x_{n+1}-x^{*} \bigr\Vert ^{q}+ \alpha_{n} M, \end{aligned}$$

where $c>0$ and $M=\sup_{n\geq1}\{q\|f(x^{*})-x^{*}\|\|x_{n+1}-x^{*}\|^{q-1},(1-\rho)\|x_{n}-x^{*}\|^{q}\}<\infty$. From $(C1)$ and $(C2)$ we get that

21 $$\begin{aligned} \lim_{n\to\infty} \Vert x_{n}-S_{n}x_{n} \Vert =0. \end{aligned}$$

We observe that

$$\begin{aligned} &\sup_{x\in \{x_{n}\}} \Vert S_{n+1}x-S_{n}x \Vert \\ &\quad =\sup_{x\in \{x_{n}\}} \bigl\Vert (1-\theta_{n+1})x+ \theta_{n+1}T_{n+1}x-(1-\theta_{n})x- \theta_{n}T_{n}x \bigr\Vert \\ &\quad \leq \vert \theta_{n+1}-\theta_{n} \vert \sup _{x\in \{x_{n}\}} \Vert x \Vert +\theta_{n+1}\sup _{x\in \{x_{n}\}} \Vert T_{n+1}x-T_{n}x \Vert + \vert \theta_{n+1}-\theta_{n} \vert \sup _{x\in \{x_{n}\}} \Vert T_{n}x \Vert \\ &\quad \leq \vert \theta_{n+1}-\theta_{n} \vert \Bigl(\sup _{x\in \{x_{n}\}} \Vert x \Vert +\sup_{x\in \{x_{n}\}} \Vert T_{n}x \Vert \Bigr)+\sup_{x\in\{x_{n}\}} \Vert T_{n+1}x-T_{n}x \Vert . \end{aligned}$$

Since $\{T_{n}\}_{n=1}^{\infty}$ satisfies the $AKTT$-condition and $\sum_{n=1}^{\infty}|\theta_{n+1}-\theta_{n}|<\infty$, we have

$$ \sum_{n=1}^{\infty}\sup_{x\in \{x_{n}\}} \Vert S_{n+1}x-S_{n}x \Vert < \infty, $$

that is, $\{S_{n}\}_{n=1}^{\infty}$ satisfies the *AKTT*-condition. From this we can define the nonexpansive mapping $S:C\to C$ by $Sx=\lim_{n\to\infty}S_{n}x$ for all $x\in C$. Since $\{\theta_{n}\}$ is bounded, there exists a subsequence $\{\theta_{n_{i}}\}$ of $\{\theta_{n}\}$ such that $\theta_{n_{i}}\to \theta$ as $i\to\infty$. It follows that

$$ Sx=\lim_{i\to\infty}S_{n_{i}}x=\lim_{i\to\infty} \bigl[(1-\theta_{n_{i}})x+\theta_{n_{i}}T_{n_{i}}x\bigr]=(1- \theta)x+\theta Tx,\quad x\in C. $$

This shows that $F(S)=F(T)=\bigcap_{n=1}^{\infty}F(T_{n}):=\Omega$. By (21) and Lemma 2.10 we have

22 $$\begin{aligned} \Vert x_{n}-Sx_{n} \Vert \leq& \Vert x_{n}-S_{n}x_{n} \Vert + \Vert S_{n}x_{n}-Sx_{n} \Vert \\ \leq& \Vert x_{n}-S_{n}x_{n} \Vert +\sup _{x\in\{x_{n}\}} \Vert S_{n}x-Sx \Vert \to0\quad \text{as }n\to\infty. \end{aligned}$$

Let $\{z_{t}\}$ be a sequence defined by

$$\begin{aligned} z_{t}=f(z_{t})+(1-t)Sz_{t},\quad t\in(0,1). \end{aligned}$$

From Lemma 2.2(i) we know that $\{x_{t}\}$ converges strongly to $x^{*}=Q(f)$, which solves the variational inequalities

$$\begin{aligned} \bigl\langle (I-f)Q(f),j_{q}\bigl(Q(f)-z\bigr)\bigr\rangle \leq0,\quad z\in \Omega. \end{aligned}$$

Moreover, we obtain that

23 $$\begin{aligned} \limsup_{n\to\infty}\bigl\langle f\bigl(x^{*} \bigr)-x^{*},j_{q}\bigl(x_{n}-x^{*}\bigr)\bigr\rangle \leq0. \end{aligned}$$

Note that

$$\begin{aligned} \Vert S_{n}z_{n}-x_{n} \Vert \leq& \Vert S_{n}z_{n}-S_{n}x_{n} \Vert + \Vert S_{n}x_{n}-x_{n} \Vert \\ \leq& \Vert z_{n}-x_{n} \Vert + \Vert S_{n}x_{n}-x_{n} \Vert \\ =&(1-s_{n}) (1-\beta_{n}) \Vert S_{n}x_{n}-x_{n} \Vert + \Vert S_{n}x_{n}-x_{n} \Vert \\ \leq&2 \Vert x_{n}-S_{n}x_{n} \Vert . \end{aligned}$$

From (21), we get that

24 $$\begin{aligned} \lim_{n\to\infty} \Vert S_{n}z_{n}-x_{n} \Vert =0. \end{aligned}$$

It follows that

25 $$\begin{aligned} &\Vert x_{n+1}-x_{n} \Vert \\ &\quad \leq \bigl\Vert \alpha_{n}\bigl(f(x_{n})-x_{n} \bigr)+(1-\alpha_{n}) (S_{n}z_{n}-x_{n}) \bigr\Vert \\ &\quad \leq \alpha_{n} \bigl\Vert f(x_{n})-x_{n} \bigr\Vert +(1-\alpha_{n}) \Vert S_{n}z_{n}-x_{n} \Vert \to0\quad \text{as } n\to\infty. \end{aligned}$$

We also have

26 $$\begin{aligned} \limsup_{n\to\infty}\bigl\langle f\bigl(x^{*} \bigr)-x^{*},j_{q}\bigl(x_{n+1}-x^{*}\bigr)\bigr\rangle \leq0. \end{aligned}$$

Again from (20), we have

27 $$\begin{aligned} & \bigl\Vert x_{n+1}-x^{*} \bigr\Vert ^{q} \end{aligned}$$

28 $$\begin{aligned} &\quad \leq \bigl(1-(1-\rho)\alpha_{n}\bigr) \bigl\Vert x_{n}-x^{*} \bigr\Vert ^{q}+q\alpha_{n}\bigl\langle f\bigl(x^{*}\bigr)-x^{*},j_{q}\bigl(x_{n+1}-x^{*}\bigr)\bigr\rangle . \end{aligned}$$

Apply Lemma 2.7 and (26) to (27), we obtain that $x_{n}\to x^{*}$ as $n\to\infty$.

*Case 2*. There exists a subsequence $\{n_{i}\}$ of $\{n\}$ such that

$$\begin{aligned} \bigl\Vert x_{n_{i}}-x^{*} \bigr\Vert \leq \bigl\Vert x_{n_{i+1}}-x^{*} \bigr\Vert \end{aligned}$$

for all $i\in\mathbb{N}$. By Lemma 2.8, there exists a nondecreasing sequence $\{m_{k}\}\subset\mathbb{N}$ such that $m_{k}\to\infty$ as $k\to\infty$ and

29 $$\begin{aligned} \bigl\Vert x_{m_{k}}-x^{*} \bigr\Vert \leq \bigl\Vert x_{{m_{k}}+1}-x^{*} \bigr\Vert \quad \text{and}\quad \bigl\Vert x_{k}-x^{*} \bigr\Vert \leq \bigl\Vert x_{{m_{k}}+1}-x^{*} \bigr\Vert \end{aligned}$$

for all $k\in\mathbb{N}$. From (20) we have

$$\begin{aligned} &(1-\alpha_{m_{k}})\beta_{m_{k}}(1-\beta_{m_{k}}) (1-s_{m_{k}})c \Vert x_{m_{k}}-S_{m_{k}}x_{m_{k}} \Vert ^{q} \\ &\quad \leq \bigl\Vert x_{m_{k}}-x^{*} \bigr\Vert ^{q}- \bigl\Vert x_{{m_{k}}+1}-x^{*} \bigr\Vert ^{q}+\alpha_{m_{k}} M \\ &\quad \leq \alpha_{m_{k}} M, \end{aligned} $$

where $c>0$ and $M<\infty$. This implies by $(C1)$ and $(C2)$ that

30 $$\begin{aligned} \Vert x_{m_{k}}-S_{m_{k}}x_{m_{k}} \Vert \to0\quad \text{as }k\to\infty. \end{aligned}$$

Since

$$\begin{aligned} &\sup_{x\in \{x_{m_{k}}\}} \Vert S_{{m_{k}}+1}x-S_{m_{k}}x \Vert \\ &\quad =\sup_{x\in \{x_{m_{k}}\}} \bigl\Vert (1-\theta_{{m_{k}}+1})x+ \theta_{{m_{k}}+1}T_{{m_{k}}+1}x-(1-\theta_{m_{k}})x- \theta_{m_{k}}T_{m_{k}}x \bigr\Vert \\ &\quad \leq \vert \theta_{{m_{k}}+1}-\theta_{m_{k}} \vert \sup _{x\in \{x_{m_{k}}\}} \Vert x \Vert +\theta_{{m_{k}}+1}\sup _{x\in \{x_{m_{k}}\}} \Vert T_{{m_{k}}+1}x-T_{m_{k}}x \Vert \\ &\qquad {}+ \vert \theta_{{m_{k}}+1}-\theta_{m_{k}} \vert \sup _{x\in \{x_{m_{k}}\}} \Vert T_{m_{k}}x \Vert \\ &\quad \leq \vert \theta_{{m_{k}}+1}-\theta_{m_{k}} \vert \Bigl(\sup _{x\in \{x_{m_{k}}\}} \Vert x \Vert +\sup_{x\in \{x_{m_{k}}\}} \Vert T_{m_{k}}x \Vert \Bigr)+\sup_{x\in \{x_{m_{k}}\}} \Vert T_{{m_{k}}+1}x-T_{m_{k}}x \Vert < \infty, \end{aligned}$$

that is, $\{S_{m_{k}}\}_{k=1}^{\infty}$ satisfies the $AKTT$-condition. Then, by (30) and Lemma 2.10, we get that

31 $$\begin{aligned} & \Vert x_{m_{k}}-Sx_{m_{k}} \Vert \\ &\quad \leq \Vert x_{m_{k}}-S_{m_{k}}x_{m_{k}} \Vert + \Vert S_{m_{k}}x_{m_{k}}-Sx_{m_{k}} \Vert \\ &\quad \leq \Vert x_{m_{k}}-S_{m_{k}}x_{m_{k}} \Vert +\sup _{x\in\{x_{m_{k}}\}} \Vert S_{m_{k}}x-Sx \Vert \to0\quad \text{as }k\to\infty. \end{aligned}$$

By the same argument as in Case 1, we can show that

32 $$\begin{aligned} \limsup_{k\to\infty}\bigl\langle f\bigl(x^{*}\bigr)-x^{*},j \bigl(x_{{m_{k}}}-x^{*}\bigr)\bigr\rangle \leq0. \end{aligned}$$

It follows from (31) that

$$\begin{aligned} \Vert S_{m_{k}}z_{m_{k}}-x_{m_{k}} \Vert \leq& \Vert S_{m_{k}}z_{m_{k}}-S_{m_{k}}x_{m_{k}} \Vert + \Vert S_{m_{k}}x_{m_{k}}-x_{m_{k}} \Vert \\ \leq& \Vert z_{m_{k}}-x_{m_{k}} \Vert + \Vert S_{m_{k}}x_{m_{k}}-x_{m_{k}} \Vert \\ =&(1-s_{m_{k}}) (1-\beta_{m_{k}}) \Vert S_{m_{k}}x_{m_{k}}-x_{m_{k}} \Vert + \Vert S_{m_{k}}x_{m_{k}}-x_{m_{k}} \Vert \\ \leq&2 \Vert x_{m_{k}}-S_{m_{k}}x_{m_{k}} \Vert \to0 \quad \text{as }k\to\infty, \end{aligned}$$

and hence

$$\begin{aligned} \Vert x_{{m_{k}}+1}-x_{m_{k}} \Vert \leq& \bigl\Vert \alpha_{m_{k}}\bigl(f(x_{m_{k}})-x_{m_{k}}\bigr)+(1- \alpha_{m_{k}}) (S_{m_{k}}z_{m_{k}}-x_{m_{k}}) \bigr\Vert \\ \leq&\alpha_{m_{k}} \bigl\Vert f(x_{m_{k}})-x_{m_{k}} \bigr\Vert +(1-\alpha_{m_{k}}) \Vert S_{m_{k}}z_{m_{k}}-x_{m_{k}} \Vert \to0\quad \text{as }k\to\infty. \end{aligned}$$

Then, we also have

33 $$\begin{aligned} \limsup_{k\to\infty}\bigl\langle f\bigl(x^{*} \bigr)-x^{*},j_{q}\bigl(x_{{m_{k}}+1}-x^{*}\bigr)\bigr\rangle \leq0. \end{aligned}$$

Again from (27) we have

34 $$\begin{aligned} & \bigl\Vert x_{{m_{k}}+1}-x^{*} \bigr\Vert ^{q} \\ &\quad \leq \bigl(1-(1-\rho)\alpha_{m_{k}}\bigr) \bigl\Vert x_{m_{k}}-x^{*} \bigr\Vert ^{q}+q\alpha_{m_{k}}\bigl\langle f\bigl(x^{*}\bigr)-x^{*},j_{q}\bigl(x_{{m_{k}}+1}-x^{*}\bigr)\bigr\rangle , \end{aligned}$$

which implies that

35 $$\begin{aligned} (1-\rho)\alpha_{m_{k}} \bigl\Vert x_{m_{k}}-x^{*} \bigr\Vert ^{q} \leq& \bigl\Vert x_{m_{k}}-x^{*} \bigr\Vert ^{q}- \bigl\Vert x_{{m_{k}}+1}-x^{*} \bigr\Vert ^{q} \\ &{}+q\alpha_{m_{k}}\bigl\langle f\bigl(x^{*}\bigr)-x^{*},j_{q} \bigl(x_{{m_{k}}+1}-x^{*}\bigr)\bigr\rangle \\ \leq&q\alpha_{m_{k}}\bigl\langle f\bigl(x^{*}\bigr)-x^{*},j_{q} \bigl(x_{{m_{k}}+1}-x^{*}\bigr)\bigr\rangle . \end{aligned}$$

Since $\alpha_{m_{k}}>0$, we get $\lim_{k\to\infty}\|x_{m_{k}}-x^{*}\|=0$. So, we have

$$\begin{aligned} \bigl\Vert x_{k}-x^{*} \bigr\Vert \leq& \bigl\Vert x_{{m_{k}}+1}-x^{*} \bigr\Vert \\ =& \bigl\Vert x_{m_{k}}-x^{*} \bigr\Vert + \bigl\Vert x_{{m_{k}}+1}-x^{*} \bigr\Vert - \bigl\Vert x_{m_{k}}-x^{*} \bigr\Vert \\ \leq& \bigl\Vert x_{m_{k}}-x^{*} \bigr\Vert + \Vert x_{{m_{k}}+1}-x_{m_{k}} \Vert \to0\quad \text{as }k\to\infty, \end{aligned}$$

which implies that $x_{k}\to x^{*}$ as $k\to\infty$. This completes the proof. □

Applying Theorem 3.1 to a 2-uniformly smooth Banach space, we obtain the following result.

### Corollary 3.2

*Let* *C* *be a nonempty closed convex subset of a real uniformly convex and* 2-*uniformly smooth Banach space* *E*. *Let* $f\in\Pi_{C}$ *with coefficient* $\rho\in(0,1)$, *and let* $\{T_{n}\}_{n=1}^{\infty}:C\to C$ *be a family of* *λ*-*strict pseudo*-*contractions such that* $\Omega:=\bigcap_{n=1}^{\infty}F(T_{n})\neq\emptyset$. *For all* $x\in C$, *define the mapping* $S_{n}x=(1-\theta)x+\theta T_{n}x$, *where* $0<\theta\leq\delta$, $\delta=\min \{1,\frac{\lambda}{K^{2}} \}$, *and* $\sum_{n=1}^{\infty}|\theta_{n+1}-\theta_{n}|<\infty$. *For given* $x_{1}\in C$, *let* $\{x_{n}\}$ *be a sequence generated by*

36 $$ \textstyle\begin{cases} \bar{x}_{n+1}=\beta_{n}x_{n}+(1-\beta_{n})S_{n}x_{n},\\ x_{n+1}=\alpha_{n} f(x_{n})+(1-\alpha_{n})S_{n}(t_{n}x_{n}+(1-t_{n})\bar{x}_{n+1}),\quad n\geq1, \end{cases} $$

*where* $\{\alpha_{n}\}$, $\{\beta_{n}\}$, *and* $\{t_{n}\}$ *are sequences in* $(0,1)$ *satisfying the conditions* $(C1)$ *and* $(C2)$ *of Theorem* 3.1. *Suppose in addition that* $(\{T_{n}\}_{n=1}^{\infty},T)$ *satisfies the* $AKTT$-*condition*. *Then* $\{x_{n}\}$ *converges strongly to* $x^{*}=Q(f)\in \Omega$, *which solves the variational inequality*

37 $$\begin{aligned} \bigl\langle (I-f)Q(f),j\bigl(Q(f)-z\bigr)\bigr\rangle \leq0,\quad \forall z\in \Omega, \end{aligned}$$

*where* *Q* *is a sunny nonexpansive retraction of* *C* *onto* Ω.

Utilizing the fact that a Hilbert space *H* is uniformly convex and 2-uniformly smooth with the best smooth constant $\kappa_{2}=1$, we obtain the following result.

### Corollary 3.3

*Let* *C* *be a nonempty closed convex subset of a Hilbert space* *H*. *Let* $f\in\Pi_{C}$ *with coefficient* $\rho\in(0,1)$, *and let* $\{T_{n}\}_{n=1}^{\infty}:C\to C$ *be a family of* *λ*-*strict pseudo*-*contractions with* $\lambda\in[0,1)$ *such that* $\Omega:=\bigcap_{n=1}^{\infty}F(T_{n})\neq\emptyset$. *For all* $x\in C$, *define the mapping* $S_{n}x=(1-\theta_{n})x+\theta_{n} T_{n}x$, *where* $0<\theta_{n}\leq\delta$, $\delta=\min \{1,2\lambda \}$, *and* $\sum_{n=1}^{\infty}|\theta_{n+1}-\theta_{n}|<\infty$. *For given* $x_{1}\in C$, *let* $\{x_{n}\}$ *be a sequence generated by*

38 $$ \textstyle\begin{cases} \bar{x}_{n+1}=\beta_{n}x_{n}+(1-\beta_{n})S_{n}x_{n},\\ x_{n+1}=\alpha_{n} f(x_{n})+(1-\alpha_{n})S_{n}(t_{n}x_{n}+(1-t_{n})\bar{x}_{n+1}),\quad n\geq1, \end{cases} $$

*where* $\{\alpha_{n}\}$, $\{\beta_{n}\}$, *and* $\{t_{n}\}$ *are sequences in* $(0,1)$ *satisfying conditions* $(C1)$ *and* $(C2)$ *of Theorem* 3.1. *Suppose*, *in addition*, *that* $(\{T_{n}\}_{n=1}^{\infty},T)$ *satisfies the* $AKTT$-*condition*. *Then* $\{x_{n}\}$ *converges strongly to* $x^{*}=P(f)\in \Omega$, *which solves the variational inequality*

39 $$\begin{aligned} \bigl\langle (I-f)P(f),P(f)-z\bigr\rangle \leq0,\quad z\in \Omega, \end{aligned}$$

*where* *P* *is a metric projection of* *C* *onto* Ω.

## Application

### The generalized viscosity explicit rules for convex combination of family of mappings

In this subsection, we apply our main result to convex combination of a countable family of strict pseudo-contractions. The following lemmas can be found in [36, 37].

#### Lemma 4.1

([36, 37])

*Let* *C* *be a closed convex subset of a smooth Banach space* *E*. *Suppose that* $\{T_{n}\}_{n=1}^{\infty}:C\to C$ *is a family of* *λ*-*strictly pseudo*-*contractive mappings with* $\bigcap_{n=1}^{\infty}F(T_{n})\neq\emptyset$ *and* $\{\mu_{n}\}_{n=1}^{\infty}$ *is a real sequence in* $(0, 1)$ *such that* $\sum_{n=1}^{\infty}\mu_{n}=1$. *Then the following conclusions hold*:

(i) *A mapping* $G:C\to E$ *defined by* $G:=\sum_{n=1}^{\infty}\mu_{n} T_{n}$ *is a* *λ*-*strictly pseudocontractive mapping*.
(ii) $F(G)=\bigcap_{n=1}^{\infty}F(T_{n})$.

#### Lemma 4.2

([37])

*Let* *C* *be a closed convex subset of a smooth Banach space* *E*. *Suppose that* $\{T_{k}\}_{k=1}^{\infty}:C\to C$ *is a countable family of* *λ*-*strictly pseudocontractive mappings with* $\bigcap_{k=1}^{\infty}F(S_{k})\neq\emptyset$. *For all* $n\in \mathbb{N}$, *define* $S_{n} : C \to C$ *by* $S_{n}x:=\sum_{k=1}^{n}\mu_{n}^{k}T_{k}x$ *for all* $x\in C$, *where* $\{\mu_{n}^{k}\}$ *is a family of nonnegative numbers satisfying the following conditions*:

(i) $\sum_{k=1}^{n}\mu_{n}^{k}=1$ *for all* $n\in \mathbb{N}$;
(ii) $\mu^{k}:=\lim_{n\to\infty}\mu_{n}^{k}>0$ *for all* $k\in \mathbb{N}$;
(iii) $\sum_{n=1}^{\infty}\sum_{k=1}^{n}|\mu_{n+1}^{k}-\mu_{n}^{k}|<\infty$.

*Then*:

1. *Each* $T_{n}$ *is a* *λ*-*strictly pseudocontractive mapping*.
2. $\{T_{n}\}$ *satisfies the AKTT*-*condition*.
3. *If* $T : C\to C$ *is defined by* $Tx=\sum_{k=1}^{\infty}\mu^{k}S_{k}x$ *for all* $x\in C$,

*then*, $Tx=\lim_{n\to\infty}T_{n}x$ *and* $F(T)=\bigcap_{n=1}^{\infty}F(T_{n})=\bigcap_{k=1}^{\infty}F(S_{k})$.

Using Theorem 3.1 and Lemmas 4.1 and 4.2, we obtain the following result.

#### Theorem 4.3

*Let* *C* *be a nonempty closed convex subset of a real uniformly convex and* *q*-*uniformly smooth Banach space* *E*. *Let* $f\in\Pi_{C}$ *with coefficient* $\rho\in(0,1)$, *and let* $\{T_{k}\}_{k=1}^{\infty}:C\to C$ *be a countable family of* $\lambda_{k}$-*strict pseudo*-*contractions with* $\inf\{{\lambda_{k}}:k\in\mathbb{N}\}=\lambda>0$. *For all* $x\in C$, *define a mapping* $S_{n}x:=(1-\theta_{n})x+\theta_{n} \sum_{k=1}^{n}\mu_{n}^{k}T_{k}x$ *such that* $\Omega:=\bigcap_{k=1}^{\infty}F(T_{k})\neq\emptyset$, *where* $0<\theta_{n}\leq\delta$, $\delta=\min \{1, (\frac{q\lambda}{\kappa_{q}} )^{\frac{1}{q-1}} \}$, *and* $\sum_{n=1}^{\infty}|\theta_{n+1}-\theta_{n}|<\infty$. *For given* $x_{1}\in C$, *let* $\{x_{n}\}$ *be a sequence generated by*

40 $$ \textstyle\begin{cases} \bar{x}_{n+1}=\beta_{n}x_{n}+(1-\beta_{n})S_{n}x_{n},\\ x_{n+1}=\alpha_{n} f(x_{n})+(1-\alpha_{n})S_{n}(t_{n}x_{n}+(1-t_{n})\bar{x}_{n+1}),\quad n\geq1, \end{cases} $$

*where* $\{\alpha_{n}\}$, $\{\beta_{n}\}$, *and* $\{t_{n}\}$ *are sequences in* $(0,1)$ *satisfy conditions* $(C1)$ *and* $(C2)$ *of Theorem* 3.1, *and* $\{\mu_{n}^{k}\}$ *is a real sequence satisfying* (i)–(iii) *of Lemma* 4.2. *Then* $\{x_{n}\}$ *converges strongly to a* $x^{*}\in\Omega$.

### The generalized viscosity explicit rules for zeros of accretive operators

In this subsection, we apply our main result to problem of finding a zero of an accretive operator. An operator $A\subset E\times E$ is said to be accretive if for all $(x_{1}, y_{1})$ and $(x_{2}, y_{2})\in A$, there exists $j_{q}\in J_{q}(x_{1}-x_{2})$ such that $\langle y_{1}-y_{2},j_{q}\rangle\geq0$. An operator *A* is said to satisfy the range condition if $\overline {D(A)}=R(I + \lambda A)$ for all $\lambda> 0$, where $D(A)$ is the domain of *A*, $R(I +\lambda A)$ is the range of $I +\lambda A$, and $\overline{D(A)}$ is the closure of $D(A)$. If *A* is an accretive operator that satisfies the range condition, then we can defined a single-valued mapping $J_{\lambda}^{A}:R(I+\lambda A)\to D(A)$ by $J_{\lambda}=(I+\lambda A)^{-1}$, which is called the *resolvent* of *A*. We denote $A^{-1}0$ by the set of zeros of *A*, that is, $A^{-1}0=\{x\in D(A):0\in Ax\}$. It is well known that $J_{\lambda}$ is nonexpansive and $F(J_{\lambda})=A^{-1}0$ (see [38]). We also know the following [39]: For all $\lambda,\mu>0$ and $x\in R(I+\lambda A)\cap R(I+\mu A)$, we have

$$\begin{aligned} \Vert J_{\lambda}x-J_{\mu}x \Vert \leq\frac{ \vert \lambda-\mu \vert }{\lambda} \Vert x-J_{\lambda}x \Vert . \end{aligned}$$

#### Lemma 4.4

([34])

*Let* *C* *be a nonempty closed convex subset of a Banach space* *E*. *Let* $A\subset E\times E$ *be an accretive operator such that* $A^{-1}0\neq\emptyset$, *which satisfies the condition* $\overline{D(A)}\subset C\subset \bigcap_{\lambda>0}R(I+\lambda A)$. *Suppose that* $\{\lambda_{n}\}\subset (0,\infty)$ *such that* $\inf\{\lambda_{n}:n\in\mathbb{N}\}>0$ *and* $\sum_{n=1}^{\infty}|\theta_{n+1}-\theta_{n}|<\infty$. *Then*, $\{J_{\lambda_{n}}\}$ *satisfies the AKTT*-*condition*. *Consequently*, *for each* $x\in C$, $\{J_{\lambda_{n}}x\}$ *converges strongly to some point of* *C*. *Moreover*, *let* $J_{\lambda}: C\to C$ *be defined by* $J_{\lambda} x=\lim_{n\to\infty}J_{\lambda_{n}}x$ *for all* $x\in C$ *and* $F(J_{\lambda})=\bigcap_{n=1}^{\infty}F(J_{\lambda_{n}})$, *where* $\lambda_{n}\to \lambda$ *as* $n\to\infty$. *Then*, $\lim_{n\to\infty}\sup_{x\in C}\|J_{\lambda} x-J_{\lambda_{n}}x\|=0$.

Utilizing Theorem 3.1 and and Lemma 4.4, we obtain the following result.

#### Theorem 4.5

*Let* *C* *be a nonempty closed convex subset of a* *q*-*uniformly smooth Banach space* *E*. *Let* $f\in\Pi_{C}$ *with coefficient* $\rho\in(0,1)$ *and let* $A\subset E\times E$ *be an accretive operator such that* $A^{-1}0\neq\emptyset$ *which satisfies the condition* $\overline{D(A)}\subset C\subset \bigcap_{\lambda>0}R(I+\lambda A)$. *Suppose that* $\{\lambda_{n}\}\subset (0,\infty)$ *is such that* $\inf\{\lambda_{n}:n\in\mathbb{N}\}>0$ *and* $\sum_{n=1}^{\infty}|\lambda_{n+1}-\lambda_{n}|<\infty$. *For given* $x_{1}\in C$, *let* $\{x_{n}\}$ *be the sequence generated by*

41 $$ \textstyle\begin{cases} \bar{x}_{n+1}=\beta_{n}x_{n}+(1-\beta_{n})J_{\lambda_{n}}x_{n},\\ x_{n+1}=\alpha_{n} f(x_{n})+(1-\alpha_{n})J_{\lambda_{n}}(t_{n}x_{n}+(1-t_{n})\bar{x}_{n+1}),\quad n\geq1, \end{cases} $$

*where* $\{\alpha_{n}\}$, $\{\beta_{n}\}$, *and* $\{t_{n}\}$ *are sequences in* $(0,1)$ *satisfying conditions* $(C1)$ *and* $(C2)$ *of Theorem* 3.1. *Then* $\{x_{n}\}$ *converges strongly to* $x^{*}\in A^{-1}0$.

### The generalized viscosity explicit rules with weak contraction

In this subsection, we apply our main result to the viscosity approximation method with weak contraction.

#### Definition 4.6

([40–42])

Let *C* be a closed and convex subset of a real Banach space *E*. A mapping $g:C\to C$ is said to be *weakly contractive* if there exists a continuous strictly increasing function $\psi:[0,\infty)\to[0,\infty)$ with $\psi(0)=0$ and $\lim_{t\to\infty}\psi(t)=\infty$ such that

$$\begin{aligned} \bigl\Vert g(x)-g(y) \bigr\Vert \leq \Vert x-y \Vert -\psi \bigl( \Vert x-y \Vert \bigr),\quad x,y\in C. \end{aligned}$$

As a particular case, if $\psi(t)=(1-\rho)t$ for all $t\geq0$, where $\rho\in(0,1)$, then the weakly contractive mapping is contraction with coefficient *ρ*.

In 2001, Rhoades [42] first proved Banach’s contraction principle for the weakly contractive mapping in complete metric space.

#### Lemma 4.7

([42])

*Let* $(E,d)$ *be a complete metric space*, *and let* *g* *be a weakly contractive mapping on* *E*. *Then* *g* *has a unique fixed point in* *E*.

#### Lemma 4.8

([43])

*Assume that* $\{a_{n}\}$ *and* $\{b_{n}\}$ *are sequences of nonnegative real number*, *and* $\{\lambda_{n}\}$ *is a sequence of a positive real number satisfying the conditions* $\sum_{n=1}^{\infty}\lambda_{n}=\infty$ *and* $\lim_{n\to\infty}\frac{b_{n}}{\lambda_{n}}=0$. *Suppose that*

$$\begin{aligned} a_{n+1}\leq a_{n}-\lambda_{n} \psi(a_{n})+b_{n},\quad n\geq1, \end{aligned}$$

*where* $\psi(t)$ *is a continuous strictly increasing function on* $\mathbb{R}$ *with* $\psi(0)=0$. *Then*, $\lim_{n\to\infty}a_{n}=0$.

Utilizing Theorem 3.1, we obtain the following result.

#### Theorem 4.9

*Let* *C* *be a nonempty closed convex subset of a real uniformly convex and* *q*-*uniformly smooth Banach space* *E*. *Let* $g:C\to C$ *be a weak contraction*, *and let* $\{T_{n}\}_{n=1}^{\infty}:C\to C$ *be a family of* *λ*-*strict pseudo*-*contractions such that* $\Omega:=\bigcap_{n=1}^{\infty}F(T_{n})\neq\emptyset$. *For all* $x\in C$, *define the mapping* $S_{n}x=(1-\theta_{n})x+\theta_{n} T_{n}x$, *where* $0<\theta_{n}\leq\delta$, $\delta=\min \{1, (\frac{q\lambda}{\kappa_{q}} )^{\frac{1}{q-1}} \}$, *and* $\sum_{n=1}^{\infty}|\theta_{n+1}-\theta_{n}|<\infty$. *For given* $x_{1}\in C$, *let* $\{x_{n}\}$ *be the sequence generated by*

42 $$ \textstyle\begin{cases} \bar{x}_{n+1}=\beta_{n}x_{n}+(1-\beta_{n})S_{n}x_{n},\\ x_{n+1}=\alpha_{n} g(x_{n})+(1-\alpha_{n})S_{n}(t_{n}x_{n}+(1-t_{n})\bar{x}_{n+1}),\quad n\geq1, \end{cases} $$

*where* $\{\alpha_{n}\}$, $\{\beta_{n}\}$, *and* $\{t_{n}\}$ *are sequences in* $(0,1)$ *satisfy conditions* $(C1)$ *and* $(C2)$ *of Theorem* 3.1. *Suppose in addition that* $(\{T_{n}\}_{n=1}^{\infty},T)$ *satisfies the* $AKTT$-*condition*. *Then* $\{x_{n}\}$ *converges strongly to* $x^{*}\in\Omega$.

#### Proof

By the smoothness of *E* there exists a sunny nonexpansive retraction *Q* from *C* onto Ω. Moreover, $Q(g)$ is a weakly contractive mapping of *C* into itself. For all $x,y\in C$, we have

$$\begin{aligned} \bigl\Vert Q\bigl(g(x)\bigr)-Q\bigl(g(y)\bigr) \bigr\Vert \leq \bigl\Vert g(x)-g(y) \bigr\Vert \leq \Vert x-y \Vert -\psi\bigl( \Vert x-y \Vert \bigr). \end{aligned}$$

Lemma 4.7 guarantees that $Q(g)$ has a unique fixed point $x^{*}\in C$ such that $x^{*}=Q(g)$. Now, we define a sequence $\{y_{n}\}$ and $y_{1}\in C$ as follows:

$$ \textstyle\begin{cases} \bar{y}_{n+1}=\beta_{n}y_{n}+(1-\beta_{n})S_{n}y_{n},\\ y_{n+1}=\alpha_{n} g(y_{n})+(1-\alpha_{n})S_{n}(t_{n}y_{n}+(1-t_{n})\bar{y}_{n+1}),\quad n\geq1. \end{cases} $$

Then, by Theorem 3.1 with a constant $f=g(x^{*})$, we have that $\{y_{n}\}$ converges strongly to $x^{*}=Q(g))\in \Omega$. Next, we show that $x_{n}\to x^{*}$ as $n\to\infty$. Since

$$\begin{aligned} \Vert \bar{x}_{n+1}-\bar{y}_{n+1} \Vert \leq& \beta_{n} \Vert x_{n}-y_{n} \Vert +(1- \beta_{n}) \Vert S_{n}x_{n}-S_{n}y_{n} \Vert \leq \Vert x_{n}-y_{n} \Vert , \end{aligned}$$

it follows that

43 $$\begin{aligned} & \Vert x_{n+1}-y_{n+1} \Vert \\ &\quad = \bigl\Vert \alpha_{n}\bigl(g(x_{n})-g\bigl(x^{*}\bigr) \bigr)+(1-\alpha_{n}) \bigl(S_{n}\bigl(t_{n}x_{n}+(1-t_{n}) \bar{x}_{n+1}\bigr)-S_{n}\bigl(t_{n}y_{n}+(1-t_{n}) \bar{y}_{n+1}\bigr)\bigr) \bigr\Vert \\ &\quad \leq \alpha_{n} \bigl\Vert g(x_{n})-g\bigl(x^{*}\bigr) \bigr\Vert +(1-\alpha_{n}) \bigl\Vert S_{n} \bigl(t_{n}x_{n}+(1-t_{n})\bar{x}_{n+1} \bigr)-S_{n}\bigl(t_{n}y_{n}+(1-t_{n}) \bar{y}_{n+1}\bigr) \bigr\Vert \\ &\quad \leq \alpha_{n} \bigl\Vert g(x_{n})-g(y_{n}) \bigr\Vert +\alpha_{n} \bigl\Vert g(y_{n})-g\bigl(x^{*} \bigr) \bigr\Vert \\ &\qquad {}+(1-\alpha_{n}) \bigl(t_{n} \Vert x_{n}-y_{n} \Vert +(1-t_{n}) \Vert \bar{x}_{n+1}- \bar{y}_{n+1} \Vert \bigr) \\ &\quad \leq \alpha_{n} \Vert x_{n}-y_{n} \Vert - \alpha_{n}\psi\bigl( \Vert x_{n}-y_{n} \Vert \bigr)+\alpha_{n} \bigl\Vert y_{n}-x^{*} \bigr\Vert \\ &\qquad {}-\alpha_{n}\psi\bigl( \bigl\Vert y_{n}-x^{*} \bigr\Vert \bigr)+(1-\alpha_{n}) \Vert x_{n}-y_{n} \Vert \\ &\quad \leq \Vert x_{n}-y_{n} \Vert -\alpha_{n} \psi\bigl( \Vert x_{n}-y_{n} \Vert \bigr)+ \alpha_{n} \bigl\Vert y_{n}-x^{*} \bigr\Vert . \end{aligned}$$

Since $\{y_{n}\}$ converges strongly to $x^{*}$, applying Lemma 4.8 to (43), we obtain that $\lim_{n\to\infty}\|x_{n}-y_{n}\|=0$. Therefore $x_{n}\to x^{*}$. This completes the proof. □

## Numerical examples

In this section, we present a numerical example of our main result.

### Example 5.1

Let $E=\ell_{4}$ and $C=\{\mathbf{x}=(x_{1},x_{2},x_{3},x_{4},\ldots)\in \ell_{4}:x_{i}\in\mathbb{R}\text{ for }i=1,2,3,\ldots\}$ with norm $\|\mathbf{x}\|_{\ell_{4}}= (\sum_{i=1}^{\infty}|x_{i}|^{4} )^{1/4}$. Let $f:C\to C$ be the contraction defined by $f(\mathbf{x})=\frac{1}{3}\mathbf{x}$. Let $\{T_{n}\}_{n=1}^{\infty}:C\to C$ be the strictly pseudo-contractive mapping defined by

$$ T_{n}\mathbf{x}= \textstyle\begin{cases} \frac{1}{n} (1,\frac{1}{2},\frac{1}{3},\frac{1}{4},0,0,0,\ldots )-2\mathbf{x}& \text{if }\mathbf{x}\neq\mathbf{0},\\ \mathbf{0}& \text{if } \mathbf{x}=\mathbf{0}, \end{cases} $$

where $\mathbf{0}=(0,0,0,0,0,0,0,\ldots)$ is the null vector on $\ell_{4}$.

- We show that $T_{n}$ is strictly pseudo-contractive. For each $n\geq1$, if $\mathbf{x},\mathbf{y}\neq\mathbf{0}$, then
  $$\begin{aligned} \bigl\langle (I-T_{n})\mathbf{x}-(I-T_{n}) \mathbf{y},j_{2}(\mathbf{x}-\mathbf{y})\bigr\rangle &=\bigl\langle 3 \mathbf{x}-3\mathbf{y},j_{2}(\mathbf{x}-\mathbf{y})\bigr\rangle \\ &=3 \Vert \mathbf{x}-\mathbf{y} \Vert _{\ell_{4}}^{2} \\ &=\frac{1}{3} \Vert 3\mathbf{x}-3\mathbf{y} \Vert _{\ell_{4}}^{2} \\ &\geq\lambda \bigl\Vert (I-T_{n})\mathbf{x}-(I-T_{n}) \mathbf{y} \bigr\Vert _{\ell_{4}}^{2} \end{aligned} $$
  for $\lambda\leq\frac{1}{3}$. Then, we can choose $\lambda=\frac{1}{3}$. Thus, $T_{n}$ is $\frac{1}{3}$-strictly pseudo-contractive with $\bigcap_{n=1}^{\infty}F(T_{n})=\{\mathbf{0}\}$. Further, we observe that $T_{n}$ is not nonexpansive.
- We show that $(\{T_{n}\}_{n=1}^{\infty},T)$ satisfies the AKTT-condition. Since
  $$\begin{aligned} &\sup_{\mathbf{x}\in \ell_{4}} \Vert T_{n+1}\mathbf{x}-T_{n} \mathbf{x} \Vert _{\ell_{4}} \\ &\quad =\sup_{\mathbf{x}\in \ell_{4}} \biggl\Vert \frac{1}{n+1} \biggl(1, \frac{1}{2},\frac{1}{3},\frac{1}{4},0,0,0,\ldots \biggr)-2 \mathbf{x}-\frac{1}{n} \biggl(1,\frac{1}{2},\frac{1}{3}, \frac{1}{4},0,0,0,\ldots \biggr)+2\mathbf{x} \biggr\Vert _{\ell_{4}} \\ &\quad = \biggl\Vert \frac{1}{n+1} \biggl(1,\frac{1}{2}, \frac{1}{3},\frac{1}{4},0,0,0,\ldots \biggr)-\frac{1}{n} \biggl(1,\frac{1}{2},\frac{1}{3},\frac{1}{4},0,0,0,\ldots \biggr) \biggr\Vert _{\ell_{4}} \\ &\quad = \biggl(\frac{1}{n}-\frac{1}{n+1} \biggr) \biggl\Vert \biggl(1, \frac{1}{2},\frac{1}{3},\frac{1}{4},0,0,0,\ldots \biggr) \biggr\Vert _{\ell_{4}}. \end{aligned}$$
  So we have
  $$\begin{aligned} \sum_{n=1}^{\infty}\sup_{\mathbf{x}\in \ell_{4}} \Vert T_{n+1}\mathbf{x}-T_{n}\mathbf{x} \Vert _{\ell_{4}} & =\lim_{n\to\infty}\sum_{k=1}^{n} \sup_{\mathbf{x}\in \ell_{4}} \Vert T_{k+1}\mathbf{x}-T_{k} \mathbf{x} \Vert _{\ell_{4}} \\ & =\biggl\Vert \biggl(1,\frac{1}{2},\frac{1}{3}, \frac{1}{4},0,0,0,\ldots \biggr) \biggr\Vert _{\ell_{4}}< \infty, \end{aligned}$$
  *that is*, $(\{T_{n}\}_{n=1}^{\infty},T)$ satisfies the AKTT-condition, where $T:C\to C$ is defined by
  $$\begin{aligned} T\mathbf{x}=\lim_{n\to\infty}T_{n}\mathbf{x}=-2\mathbf{x}, \quad \mathbf{x}\in C. \end{aligned}$$

Since in $\ell_{4}$, $q = 2$ and $\kappa_{2}=3$, we can choose $\theta_{n}=\frac{1}{9n}+\frac{1}{9}$. Define the mapping $\{S_{n}\}_{n=1}^{\infty}:C\to C$ by

$$ S_{n}\mathbf{x}= \textstyle\begin{cases} (\frac{2}{3}-\frac{1}{3n} )\mathbf{x}+ (\frac{1}{9n^{2}}+\frac{1}{9n} ) (1,\frac{1}{2},\frac{1}{3},\frac{1}{4},0,0,0,\ldots )& \text{if } \mathbf{x}\neq\mathbf{0},\\ \mathbf{0}& \text{if }\mathbf{x}=\mathbf{0}. \end{cases} $$

Since $(\{T_{n}\}_{n=1}^{\infty},T)$ satisfies the AKTT condition, we also have that $(\{S_{n}\}_{n=1}^{\infty},S)$ satisfies the AKTT condition, where $S:C\to C$ is defined by

$$\begin{aligned} S\mathbf{x}=\lim_{n\to\infty}S_{n}\mathbf{x}= \frac{2}{3}\mathbf{x},\quad \mathbf{x}\in C. \end{aligned}$$

Then, we have $F(S)=F(T)=\bigcap_{n=1}^{\infty}F(T_{n})=\{\mathbf{0}\}$. Let $\alpha_{n}=\frac{1}{32n+1}$, $\beta_{n}=\frac{1}{100n+3}+0.32$, and $t_{n}=\frac{n}{2n+1}$. So our algorithm (16) has the following form:

44 $$ \textstyle\begin{cases} \bar{\mathbf{x}}_{n+1}= (\frac{1}{100n+3}+0.32 )\mathbf{x}_{n}+ (0.68-\frac{1}{100n+3} )S_{n}\mathbf{x}_{n},\\ \mathbf{x}_{n+1}=\frac{1}{32n+2}f(\mathbf{x}_{n})+\frac{ 32 n}{32n+1}S_{n} (\frac{n}{2n+1}\mathbf{x}_{n}+\frac{n+1}{2n+1}\bar{\mathbf{x}}_{n+1} ),\quad n\geq1. \end{cases} $$

**Figure 1:**
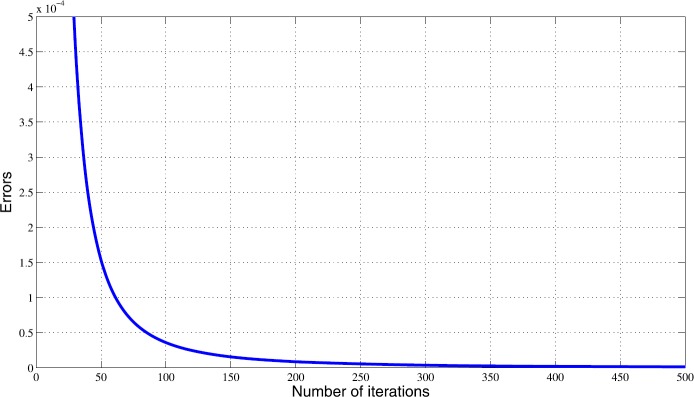
The behavior of errors

Let $\mathbf{x_{1}}=(1,-0.25,1.46,1.85,0,0,0,\ldots)$ be the initial point. Then, we obtain numerical results in Table 1 and Fig. 1.

**Table 1:** The values of the sequences $\{\mathbf{x}_{n}\}$

*n*	$\mathbf{x}_{n}$	$\|\mathbf{x}_{n+1}-\mathbf{x}_{n}\|_{\ell_{4}}$
1	(1.000000, −0.250000, 1.460000, 1.850000, 0, 0, 0,…)	1.459e+00
50	(0.007006, 0.003503, 0.002335, 0.001751, 0, 0, 0,…)	1.471e−04
100	(0.003416, 0.001708, 0.001139, 0.000854, 0, 0, 0,…)	3.531e−05
150	(0.002258, 0.001129, 0.000753, 0.000565, 0, 0, 0,…)	1.549e−05
200	(0.001687, 0.000843, 0.000562, 0.000422, 0, 0, 0,…)	8.657e−06
⋮	⋮	⋮
400	(0.000838, 0.000419, 0.000279, 0.000210, 0, 0, 0,…)	2.143e−06
450	(0.000745, 0.000372, 0.000248, 0.000186, 0, 0, 0,…)	1.692e−06
500	(0.000670, 0.000335, 0.000223, 0.000167, 0, 0, 0,…)	1.369e−06

## Conclusion

In this work, we introduce an algorithm by a generalized viscosity explicit rule for finding a common fixed point of a countable family of strictly pseudo-contractive mappings in a *q*-uniformly smooth Banach space. We obtain some strong convergence theorem for the sequence generated by the proposed algorithm under suitable conditions. However, we should like remark the following:

1. We extend the results of Ke and Ma [21] and Marino et al. [25] from a one nonexpansive mapping in Hilbert spaces to a countable family of strictly pseudo-contractive mappings in a *q*-uniformly smooth Banach space.
2. Our result is proved with a new assumption on the control conditions $\{\beta_{n}\}$ and $\{t_{n}\}$.
3. The method of proof of our result is simpler in comparison with the results of [19, 21, 44, 45]). Moreover, we remove the conditions $\sum_{n=1}^{\infty}|\alpha_{n+1}-\alpha_{n}|<\infty$ and $0<\epsilon\leq s_{n}\leq s_{n+1}<1$ in Theorem 3.1 of [21].
4. We give a numerical example that shows the efficiency and implementation of our main result in the space $\ell_{4}$, which is a uniformly convex and 2-uniformly smooth Banach space but not a Hilbert space.
